# From Hypersensitivity Pneumonitis to Lung Adenocarcinoma: A Case Report Illustrating Diagnostic Complexity

**DOI:** 10.7759/cureus.89584

**Published:** 2025-08-07

**Authors:** Umesh Kumar Pabani, Subirna Visvalingam, Katie Jones, Ahmed Sidky, Moska Rasoul

**Affiliations:** 1 Internal Medicine, Queen's Hospital, London, GBR; 2 Cardiology, Southend University Hospital, Mid and South Essex NHS Foundation Trust, Southend-on-Sea, GBR; 3 Critical Care Medicine, Southend University Hospital, Mid and South Essex NHS Foundation Trust, Southend-on-Sea, GBR; 4 Respiratory Medicine, Southend University Hospital, Mid and South Essex NHS Foundation Trust, Southend-on-Sea, GBR; 5 Acute Medicine, Southend University Hospital, Mid and South Essex NHS Foundation Trust, Southend-on-Sea, GBR

**Keywords:** adenocarcinoma lung, clinical case report, hypersensitivity pneumonitis, interstitial lung disease, non-small cell lung cancer (nsclc), pulmonary fibrosis

## Abstract

Adenocarcinoma of the lung is the most common type of lung cancer and is classified as one of the non-small cell lung cancers. It typically arises in the peripheral regions of the lungs, affecting the dense glandular tissues. Most patients diagnosed with pulmonary adenocarcinoma are current or former smokers and present with nonspecific respiratory symptoms such as a persistent cough and shortness of breath. Many also go on to develop B symptoms, including weight loss and night sweats.

We present a case of an 84-year-old Caucasian woman, a lifelong nonsmoker and teetotaller, who presented with a two-week history of dry cough, shortness of breath, and chest heaviness following receipt of her influenza vaccination. Her past medical history included hypertension (treated with amlodipine and perindopril), glaucoma, and bilateral cataracts. Initial blood tests showed normal infection and inflammatory markers. However, her chest X-ray was suggestive of pulmonary fibrosis, with fibrotic changes predominantly in the bilateral lower lung zones. She was initially treated with a tapering course of prednisolone for suspected hypersensitivity pneumonitis. Lung function tests were arranged, and a computed tomography chest scan revealed tiny centrilobular nodules in both lungs, located in peribronchovascular, perifissural, and subpleural areas. Further history revealed regular exposure to a Western Rosella bird. She denied dampness, mold, or known asbestos exposure at home. Connective tissue disease screening and avian precipitins, however, were negative.

She later presented again with worsening symptoms. Arterial blood gas analysis revealed type 1 respiratory failure. The patient was admitted to the intensive care unit and was intubated and ventilated. A repeat chest X-ray showed progressive parenchymal changes without regression. Intravenous 500 mg of methylprednisolone was started; however, she showed no improvement with it. The patient's case was discussed at the lung multidisciplinary team meeting, and a lung biopsy was recommended, which was carried out via bronchoscopy. Histopathology revealed fragmented cores of adenocarcinoma with lepidic and papillary growth patterns, mucinous type, consistent with a primary lung origin (T4N0M1a). The patient’s condition continued to deteriorate despite intensive care support, and she sadly passed away two weeks after admission.

This case underscores the need to maintain a broad differential diagnosis, particularly in patients who fail to improve with treatment. It also emphasizes the role of biopsy in resolving the diagnostic challenge, as imaging studies did not provide a clear diagnosis.

## Introduction

Lung cancer is the third most commonly diagnosed cancer overall and the second most commonly diagnosed in women [1]. It remains the leading cause of cancer-related death in the United Kingdom and worldwide. Lung adenocarcinoma, the most common subtype of lung cancer, is frequently diagnosed at a late stage due to the nonspecific nature of presenting symptoms. The most common symptoms of non-small cell lung cancer (NSCLC) include cough, chest pain, dyspnea, and weight loss [2].

Approximately 85% of lung cancers are of NSCLC origin, with the majority being adenocarcinomas arising from glandular epithelial tissue. Adenocarcinomas are strongly associated with cigarette smoking but also show increased prevalence in younger women and individuals of Asian descent. Like most lung cancers, adenocarcinomas are often asymptomatic in the early stages, and when detected early, it is frequently found as an incidental finding. However, most cases present late, contributing to a global mortality rate exceeding one million deaths annually [3].

Patients with adenocarcinoma often present with systemic "B symptoms" such as night sweats, weight loss, anorexia, cough (with or without hemoptysis), dyspnea, and fatigue [4]. However, these symptoms are nonspecific and can overlap with other common pulmonary conditions, such as interstitial pulmonary fibrosis [5]. Differentiating lung cancer from Interstitial lung disease (ILD) presents a significant diagnostic challenge on radiological imaging, as overlapping features can obscure clear identification of malignancy.

## Case presentation

We present the case of an 84-year-old Caucasian woman who attended the Same Day Emergency Care department of a busy district general hospital with a two-week history of dry cough, shortness of breath, and chest heaviness on inspiration. These symptoms developed following her annual influenza vaccination. She denied fevers, rigors, trauma, recent foreign travel, or red flag symptoms such as night sweats, weight loss, or hemoptysis. She was a lifelong nonsmoker with no known asbestos exposure. Her past medical history included hypertension, for which she was taking amlodipine 5 mg once daily and perindopril 8 mg once daily, as well as glaucoma and bilateral cataracts. She was fully independent and mobile before presentation.

**Figure 1:**
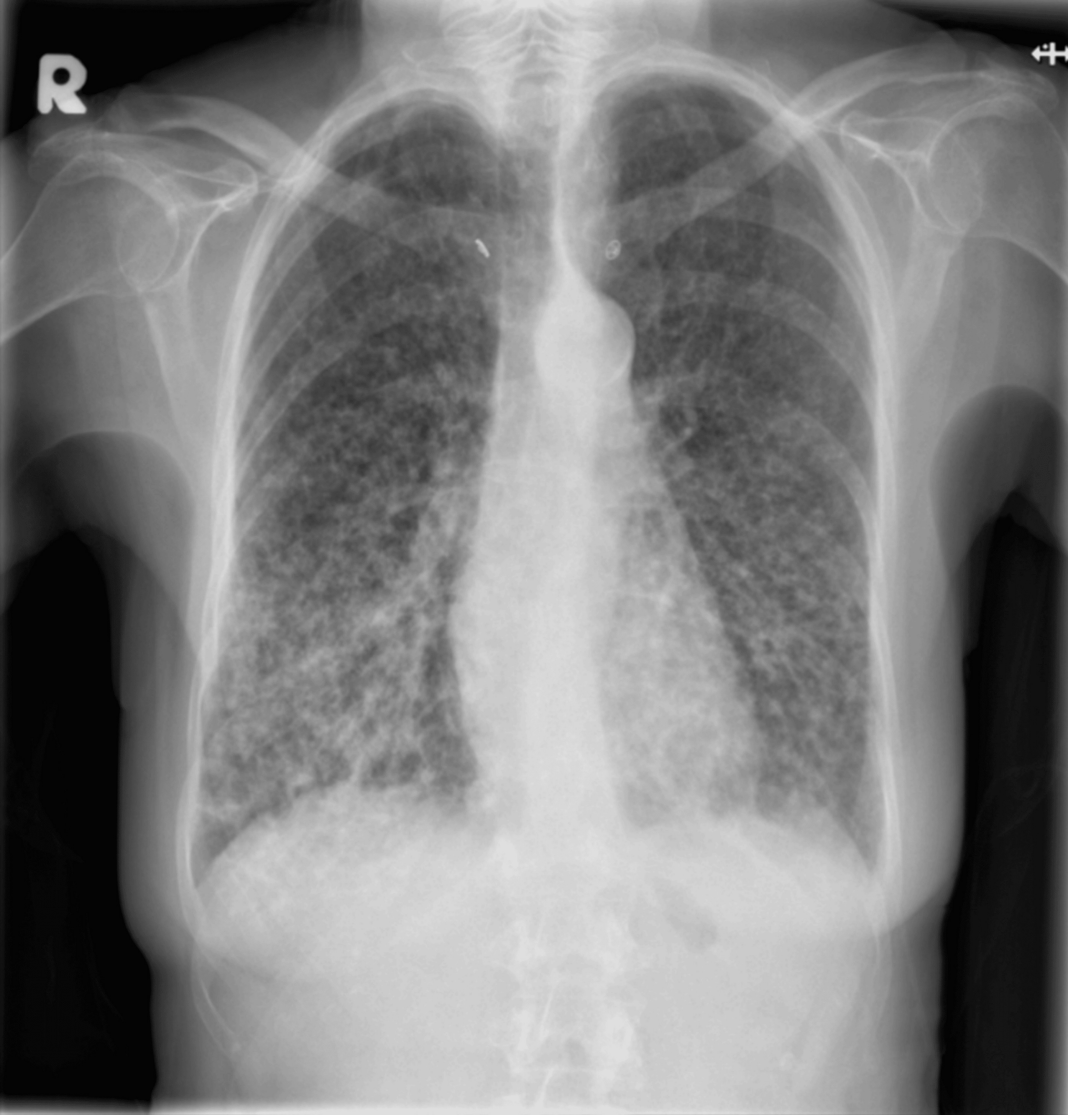
Chest X-ray on first presentation demonstrating extensive bilateral lung nodules involving all the zones with some reticulations toward the lower zones

On examination, she had bilateral lower lobe crepitations on lung auscultation and a soft ejection systolic murmur. Initial blood tests, including full blood count, clotting profile, D-dimer, urea and electrolytes, liver function tests, and C-reactive protein, were within normal limits, as shown in Table 1. Although her clinical picture was initially suggestive of a viral respiratory illness, her chest X-ray showed features concerning for pulmonary fibrosis (Figure 1). A high-resolution computed tomography (CT) scan of the chest was arranged, along with outpatient respiratory follow-up.

**Table 1:** Blood test results at the first presentation

Parameter	Result	Units	Reference range
Hemoglobin	123	g/L	115-165
Platelets	346	10^6^/L	150-400
White blood cells	8.6	10^6^/L	4.0-11.0
Neutrophils	6.44	10^6^/L	1.7-7.5
Lymphocytes	1.49	10^6^/L	1.0-4.5
Monocytes	0.61	10^6^/L	0.2-0.8
Eosinophils	0.02	10^6^/L	0.0-0.4
Basophils	0.04	10^6^/L	0.0-0.1
Sodium	139	mmol/L	133-146
Potassium	4.0	mmol/L	3.5-5.3
Creatine	59	umol/L	45-80
Urea	7.0	mmol/L	2.5-7.8
Calcium	2.48	mmol/L	2.2-2.6
D-dimer	199	Ng/mL	<243
Troponin T	12	Ng/L	<14

**Figure 2:**
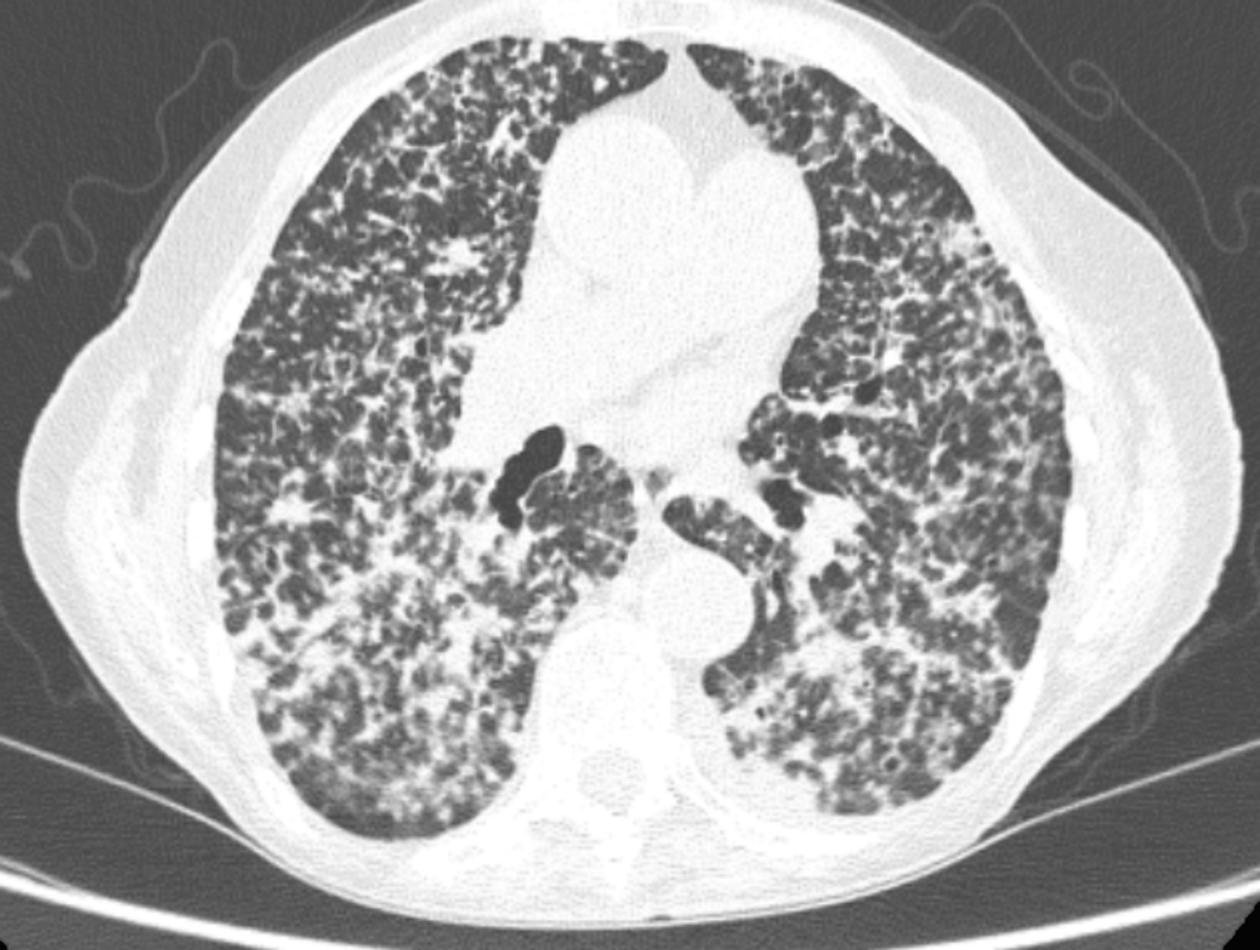
High-resolution CT of the chest, suggestive of diffuse parenchymal lung disease with numerous centrilobular nodules scattered throughout the bilateral lung parenchyma CT: computed tomography

At the respiratory clinic follow-up, the patient denied exposure to mold, damp, or asbestos; however, she regularly looked after a Western rosella bird owned by her daughter. The chest CT demonstrated numerous centrilobular nodules scattered throughout the bilateral lung parenchyma, particularly in peribronchovascular, perifissural, and subpleural regions, with a lower lobe predominance (Figure 2). The radiological findings were suggestive of diffuse parenchymal lung disease, with differential diagnoses including Langerhans cell histiocytosis, sarcoidosis, and chronic hypersensitivity pneumonitis (HP). Based on this exposure history and radiological findings, a provisional diagnosis of HP was made.

She was commenced on prednisolone 30 mg once daily and was tested for avian precipitins, which later returned negative (Table 2). Lung function tests were also performed (Table 3). Her case was discussed at a lung multidisciplinary team (MDT) meeting, where it was agreed that, although treatment for HP had been initiated, other pathologies needed to be excluded.

**Table 2:** Further respiratory workup after the initial presentation IgG: immunoglobulin G; IgE: immunoglobulin E; ANA: antinuclear antibody; ANCA: antineutrophil cytoplasmic antibody; ENA: extractable nuclear antigen

Parameter	Result	Units	Reference range
Ca125	83	kU/L	<35
*Aspergillus fumigatus*-specific IgG	32.20	mgA/L	Normal < 40
IgE	20.0	kU/L	-
ANA	<1:80	-	-
ANCA	<1:20	-	-
ENA screen	0.2	-	0.1-0.69
Budgerigar-specific IgG	5.76	mA/L	<8
Pidgeon-specific IgG	15.20	mA/L	<38

**Table 3:** Lung function tests suggesting restrictive lung pathology FEV1: forced expiratory volume in 1 second; FVC: forced vital capacity; DLCO: diffusing capacity of the lungs for carbon monoxide; KCO: transfer coefficient of carbon monoxide; RV: residual volume

Parameters	Values
FEV1	1.4 L (90% predicted)
FVC	1.69 L (87% predicted)
DLCO	35% predicted
KCO	67% predicted
RV	92% predicted

**Figure 3:**
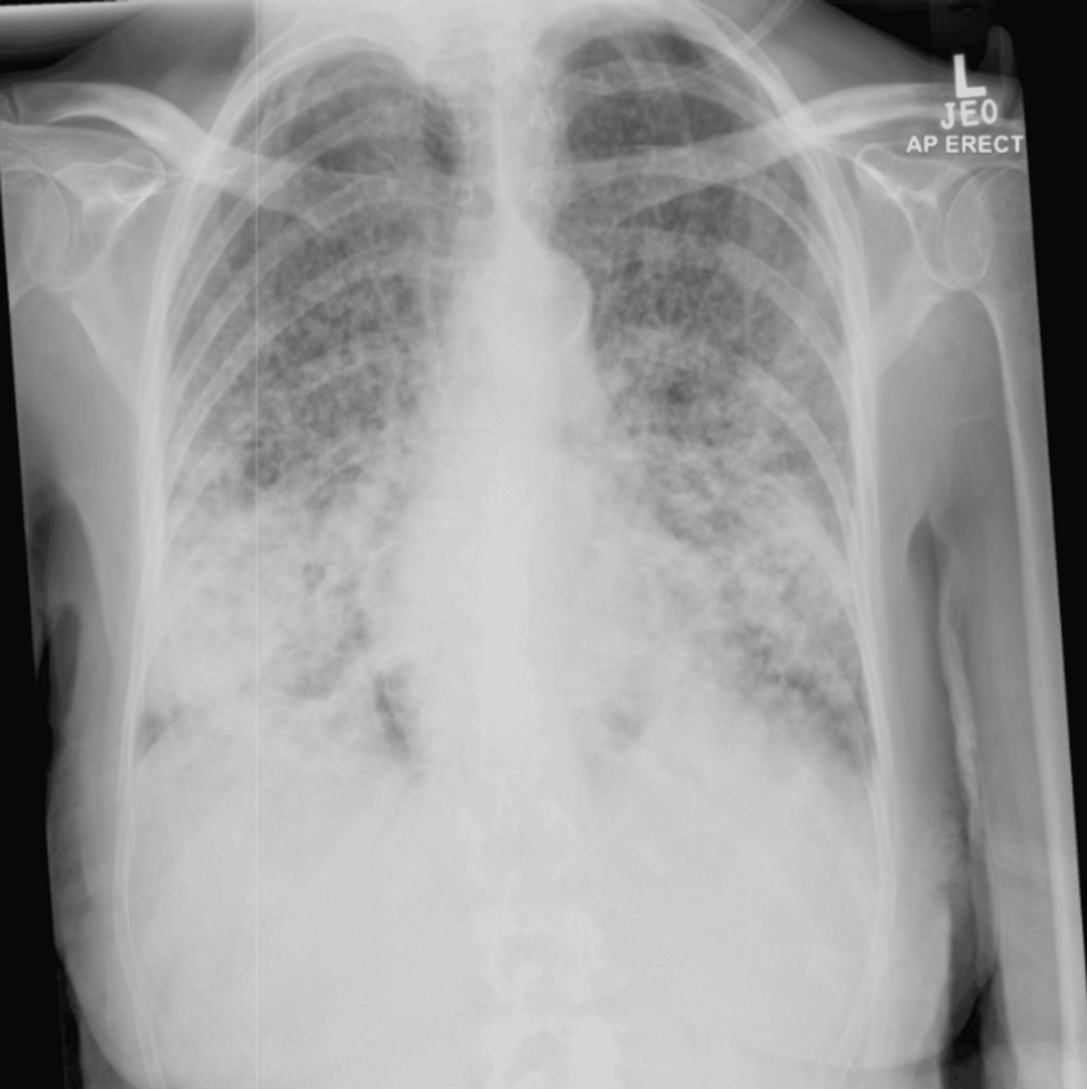
Chest X-ray two months after presentation, demonstrating worsening diffuse patchy infiltrates throughout both lungs, most concentrated within the mid to lower zones, on a background of multinodular opacity

Two months later, she re-presented with worsening dyspnea and a new oxygen requirement of 5 L/minute. Arterial blood gas analysis revealed type 1 respiratory failure, as shown in Table 4, and a repeat chest X-ray (Figure 3) showed worsening parenchymal changes. She was treated with intravenous methylprednisolone 500 mg for presumed HP exacerbation. However, her condition deteriorated, requiring intubation and mechanical ventilation in the intensive care unit. Her poor response to immunosuppressive therapy raised concern for an alternative diagnosis, including malignancy.

**Table 4:** Arterial blood gas during admission, showing type 1 respiratory failure pCO_2_: partial pressure of carbon dioxide; pO_2_: partial pressure of oxygen; HCO_3_^-^: bicarbonate; BE: base excess; sO_2_: oxygen saturation

Parameters	Values
pH	7.459
pCO_2_	4.96 KPa
pO_2_	9.65 KPa
HCO_3_^-^	26.6 mmol/L
BE	2.5
sO_2_	96.3%
Lactate	0.6 mmol/L

**Figure 4:**
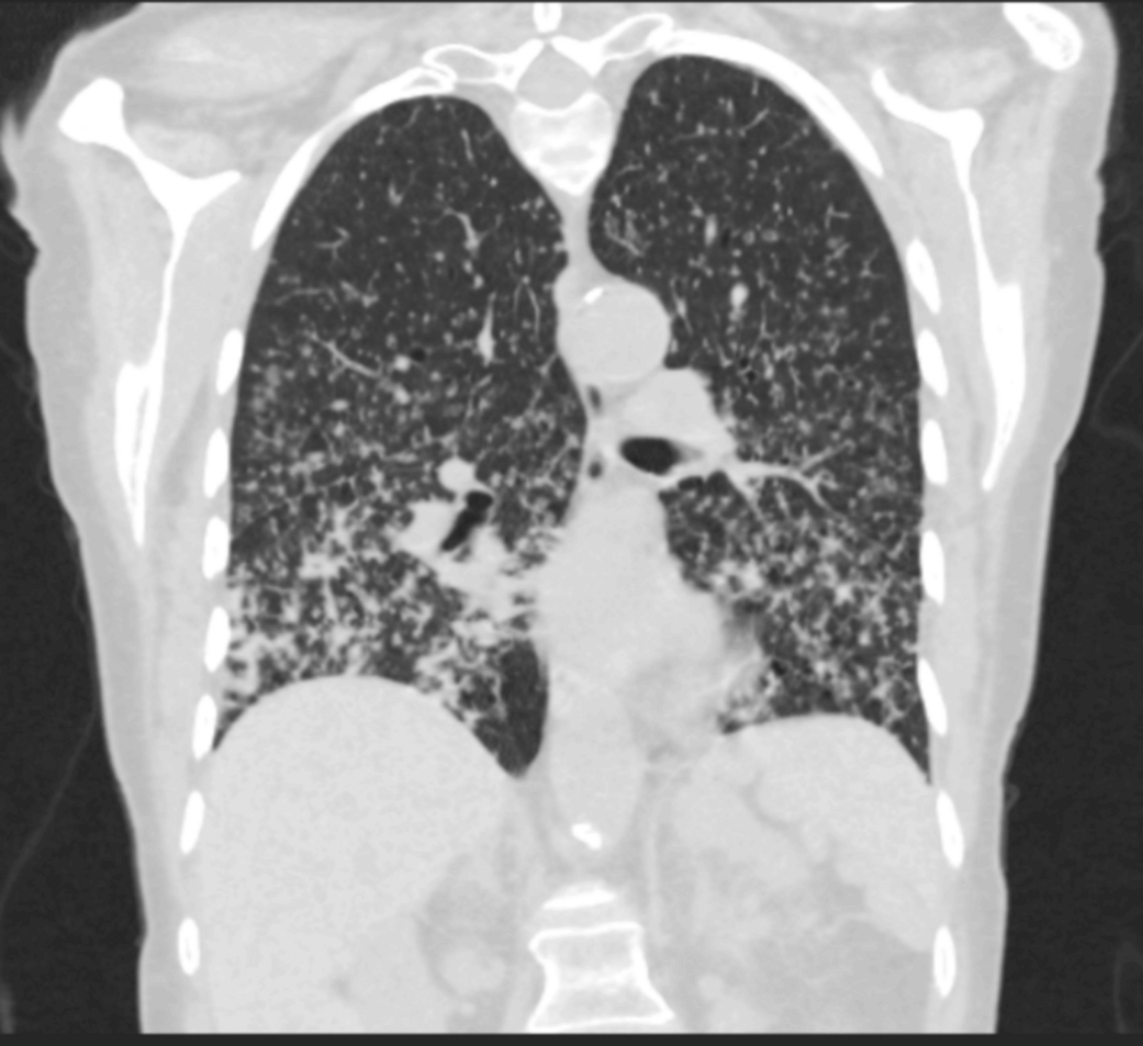
CT of the chest, demonstrating nodular opacities involving multiple lobes of both lungs CT: computed tomography

She was rediscussed at the lung MDT, which recommended obtaining tissue for histological diagnosis via bronchoscopy. Bronchoscopic biopsy revealed fragmented cores of adenocarcinoma with lepidic and papillary growth patterns, mucinous type, consistent with a primary pulmonary origin. Patient subsequently had a staging scan (CT thorax, abdomen, and pelvis), which confirmed T4N0M1a disease (cancer involving separate lobes on the ipsilateral and contralateral lungs), as shown in Figure 4.

The patient stayed in the intensive care unit for two weeks. Sadly, despite intensive care support, her condition did not improve, and she passed away two weeks after admission.

## Discussion

HP is a complex syndrome of varying intensity, clinical presentation, and natural history, caused by repeated exposure and sensitization to a wide variety of inhaled environmental antigens, leading to immune-mediated inflammation of the lung parenchyma [5]. A retrospective study of chronic HP patients found a 10.6% prevalence of lung cancer, most commonly squamous cell carcinoma, often arising adjacent to fibrotic changes in the lung tissue, suggesting that progressive fibrosis in HP may predispose to cancer development over time. ILD, including chronic HP, is increasingly recognized as a risk factor for lung cancer. This is believed to be driven by recurrent epithelial injury, chronic inflammation, genetic predisposition, and fibrotic remodeling [6,7].

In this report, we described the case of an elderly female patient with clinical symptoms and radiographic findings initially suggestive of ILD. However, due to persistent clinical deterioration despite treatment, a lung biopsy was performed, which confirmed the diagnosis of mucinous adenocarcinoma. This underscores the diagnostic complexity in differentiating between ILD and lung cancer and highlights the necessity of tissue diagnosis in uncertain or deteriorating cases.

Chest radiography remains a useful first-line tool in identifying ILDs, particularly when compared with prior imaging for progression. Common findings in idiopathic pulmonary fibrosis (IPF) include diffuse reticulonodular opacities or ground-glass changes, though these patterns are nonspecific and often overlap with other conditions, including malignancy [8]. The risk of lung cancer is increased in patients with ILD, and postmortem studies suggest a significant rate of underdiagnosis during life [9]. While chest X-rays have an estimated diagnostic accuracy of 80%, high-resolution computed tomography (HRCT) provides greater specificity and sensitivity and is now considered essential in both the diagnosis and monitoring of ILD.

Among different subtypes of ILD, IPF is more commonly associated with lung cancer than HP. Patients with IPF had a seven-fold increased risk of lung cancer compared to the general population [10]. Lung cancers in the setting of IPF often present as solid nodules; however, they may be misinterpreted as infection or fibrosis, as seen in this case [11]. This highlights the potential for missed or delayed diagnoses when relying solely on imaging. Although CT is the standard imaging modality for diagnosing and staging lung cancer, further investigations, including histopathology, are sometimes essential for accurate diagnosis and treatment planning [12].

Current guidelines do not recommend routine lung cancer screening in patients with ILD, despite their elevated risk. HRCT alone may be insufficient to differentiate fibrosis from neoplasia. Positron-emission tomography computed tomography (PET/CT) has shown improved sensitivity and specificity in detecting malignancy in patients with IPF [13]. PET/CT also allows for noninvasive characterization of disease and may detect malignancies not visible on HRCT [14].

In our patient, multiple bilateral pulmonary nodules were most prominent in the lower lobes. This is consistent with existing literature showing a predilection for adenocarcinomas to arise in peripheral and lower lung zones, especially in patients with fibrotic lung disease [15].

The role of lung biopsy in IPF remains controversial due to significant procedural risks, including pneumothorax and acute exacerbation of fibrosis, particularly when honeycomb changes are traversed [16]. In CT-guided percutaneous transthoracic needle biopsy, sensitivity and specificity can reach 90% and 84%, respectively, but with a high complication rate (12%) and nondiagnostic yield in up to 34% of cases [13]. As such, PET/CT may serve as a safer alternative in cases where biopsy is high risk or inconclusive [17].

Interestingly, PET scans have demonstrated increased FDG uptake in regions appearing normal on HRCT in patients with IPF, suggesting that metabolic imaging may detect malignancy earlier than structural imaging alone [14].

Recent research also supports the use of radiomics, a quantitative imaging approach, to stratify lung cancer risk in IPF patients using HRCT data [18]. This could enable earlier and more accurate detection and support personalized surveillance strategies in this high-risk population.

## Conclusions

This case highlights the significant diagnostic challenges in distinguishing lung adenocarcinoma from ILD, particularly in patients presenting with nonspecific respiratory symptoms and interstitial changes on imaging. Despite initial treatment for presumed HP, the patient’s clinical deterioration underscored the need for histological confirmation. This case reinforces the importance of maintaining a high index of suspicion for malignancy in atypical presentations, even in nonsmokers, and supports the role of early tissue diagnosis in improving diagnostic accuracy. Enhanced diagnostic strategies and clearer guidelines may help prevent delayed cancer diagnoses in patients with suspected ILD.

## References

[1] (2025). Office for National Statistics. Cancer registration statistics, England statistical bulletins - Office for National Statistics. https://www.ons.gov.uk/peoplepopulationandcommunity/healthandsocialcare/conditionsanddiseases/bulletins/cancerregistrationstatisticsengland/previousReleases.

[2] Ruano-Raviña A, Provencio M, Calvo de Juan V (2020). Lung cancer symptoms at diagnosis: results of a nationwide registry study. ESMO Open.

[3] The Cancer Genome Atlas Research Network (2014). Comprehensive molecular profiling of lung adenocarcinoma. Nature.

[4] Myers DJ, Wallen JM (2023). Lung Adenocarcinoma. https://www.ncbi.nlm.nih.gov/books/NBK519578/.

[5] Vasakova M, Morell F, Walsh S, Leslie K, Raghu G (2017). Hypersensitivity pneumonitis: perspectives in diagnosis and management. Am J Respir Crit Care Med.

[6] Kuramochi J, Inase N, Miyazaki Y, Kawachi H, Takemura T, Yoshizawa Y (2011). Lung cancer in chronic hypersensitivity pneumonitis. Respiration.

[7] Hamblin M, Prosch H, Vašáková M (2022). Diagnosis, course and management of hypersensitivity pneumonitis. Eur Respir Rev.

[8] Walsh SL, Devaraj A, Enghelmayer JI (2018). Role of imaging in progressive-fibrosing interstitial lung diseases. Eur Respir Rev.

[9] Abu Qubo A, Numan J, Snijder J (2022). Idiopathic pulmonary fibrosis and lung cancer: future directions and challenges. Breathe (Sheff).

[10] Hubbard R, Venn A, Lewis S, Britton J (2000). Lung cancer and cryptogenic fibrosing alveolitis. A population-based cohort study. Am J Respir Crit Care Med.

[11] Elicker BM, Kallianos KG, Henry TS (2017). The role of high-resolution computed tomography in the follow-up of diffuse lung disease: number 2 in the series "Radiology" edited by Nicola Sverzellati and Sujal Desai. Eur Respir Rev.

[12] Maconachie R, Mercer T, Navani N, McVeigh G (2019). Lung cancer: diagnosis and management: summary of updated NICE guidance. BMJ.

[13] Shin YJ, Yun G, Yoon SH (2021). Accuracy and complications of percutaneous transthoracic needle lung biopsy for the diagnosis of malignancy in patients with idiopathic pulmonary fibrosis. Eur Radiol.

[14] Win T, Thomas BA, Lambrou T (2014). Areas of normal pulmonary parenchyma on HRCT exhibit increased FDG PET signal in IPF patients. Eur J Nucl Med Mol Imaging.

[15] Liu Y, Zhu M, Geng J (2018). Incidence and radiologic-pathological features of lung cancer in idiopathic pulmonary fibrosis. Clin Respir J.

[16] Frank AJ, Dagogo-Jack I, Dobre IA (2023). Management of lung cancer in the patient with interstitial lung disease. Oncologist.

[17] Lee SH, Sung C, Lee HS (2018). Is (18)F-FDG PET/CT useful for the differential diagnosis of solitary pulmonary nodules in patients with idiopathic pulmonary fibrosis?. Ann Nucl Med.

[18] Liang CH, Liu YC, Wan YL, Yun CH, Wu WJ, López-González R, Huang WM (2021). Quantification of cancer-developing idiopathic pulmonary fibrosis using whole-lung texture analysis of HRCT images. Cancers (Basel).

